# Research on similarity test design and characteristic verification for tank fires under environmental wind conditions

**DOI:** 10.1371/journal.pone.0340275

**Published:** 2026-01-09

**Authors:** Yi Jiang, Fang Shen, Zhuo Su, Lijiao Huang

**Affiliations:** Hunan Safety Production Scientific Research Co., LTD., Changsha, China; Aalto University, FINLAND

## Abstract

Petroleum is recognized as a crucial strategic material for national sustainable development, and its safe storage, transportation, and utilization have significant impacts on the human ecological environment. In order to conduct in-depth research on the safety characteristics of oil tank fires, this study primarily employed similarity criteria to design small-scale experiments and further validated the full-scale experiments using numerical simulations. The flame characteristics, thermal physical properties, and “safe distance-time” of large-scale storage tank fires were pointed out. The results showed that with the increase of wind speed, its impact on flame height became smaller and smaller. The suitable wind speed range for the experiment was found to be between 0.97 m/s and 6.64 m/s. The flame surface area and flame volume first decreased and then increased with increasing wind speed, and there was a linear relationship between flame volume and surface area. The flame temperature decreased first and then increased. The larger the flame volume, the higher the heat release rate, and there was a linear relationship between the two. The validation experiment results showed that the temperature error and heat release rate error range of the simulation experiment were less than 5%, indicating a high reliability of the similarity experiments. Additionally, research on the “safe distance-time” relationship of the tank fires indicated that the minimum safe distance for personnel under this engineering condition was 21 m.

## 1 Introduction

Petroleum is an important energy source for sustainable development of society and the lifeblood of national economic development. It has special significance for energy reserve strategy [1,2,3]. However, this kind of fuel will occur “run, drip, leak” and other leakage phenomena during storage. Once mixed with oxygen, it is easy to cause fire after encountering open-flame. According to statistics, there are about 15–20 large-scale fuel tank fire accidents every year in the world, resulting in a large number of casualties and property losses [4,5]. Therefore, it is necessary to study the fire characteristics of such tank fuel accidents.

At present, the main methods for the study of oil tank fire are similar experiments and numerical simulation. There are currently some international standards instituted, such as the AS 4391 of Australia, JISA 4303(Japan), and GA/T999 (China). In academic research, methanol, ethanol, heptane, petrochemical oils, and other similar substances are frequently employed as common fuel sources for pool fire experiments [6,7]. Blinov and Hottel et al. studied the turbulent fire state under different fuel types and different tank sizes, and pointed out the characteristics of fuel combustion speed and flame heat transfer form [8,9]. Fabio Ferrero et al. obtained the effect of wind speed on flame height, tilt and pulsation by studying a large pool fire experiment with a diameter of 1.5 ~ 6m [10] .J. M. CHATRIS et al. studied the combustion process of pool size and fuel type under different wind speeds, and found that 2m/s was the critical wind speed that affects the combustion rate of pool fire [11]. Longhua Hu et al. studied the influence of cross airflow on the flame length and burning rate of hydrocarbon pool fires, and established a generalized model describing the flame length extension behavior [12]. Jiao Lei studied the burning rate of fuel mass under different wind speed conditions through the modified Froude number design experiment [13]. In terms of numerical simulation, Feng Zhou pointed out that wind speed will affect the flame, smoke, temperature and thermal radiation intensity of tank fire [14]. Zhuang Lei et al. conducted a numerical simulation of the combustion of a 20m diesel tank, and divided the safe distance of its thermal radiation to death, injury and disaster [15].After studying the combustion behavior of oil pool fire, Dang Xiaobei et al. pointed out the evolution law of combustion rate and flame height of oil pool fire [Dang and He, 2018].Giordano Emrys Scarponi et al. verified the safety and reliability of liquefied petroleum gas storage tanks under fire conditions according to temperature and pressure conditions through CFD software [16]. Additionally, the change of flame shape, including the flame inclination, flame height and flame shape [17], temperature and pressure is triggering by the surround wind around the tank [18]. Furthermore, the structure stability [19] and safety distant [20] of tank obtain the similar fluctuation change. In conclusion, there is a valuable insight if the change of flame shape [21] and thermal physics (temperature and the thermal radiation the size) on fire affected by the size and surround wind of tank are followed concentratively [22,23,24,25].

Although the above research has conducted in-depth exploration on the combustion characteristics of pool fires under different fuel types, tank sizes, and environmental wind conditions, and established some theoretical models and critical parameters. However, existing research mostly focuses on the impact of a single factor on fire behavior, with insufficient attention paid to the similarity criteria between small-scale experiments and actual working conditions, especially the lack of control over the experimental environment, and insufficient research on the safety of thermal radiation from tank fires on human health. Based on this, this article uses the experimental similarity criterion to conduct design experiments, quantitatively analyzing for the first time the influence of tank scale and environmental wind conditions on the flame morphology and thermophysical state changes during a fire process. The accuracy of the similarity experiment is verified through full-scale numerical simulation experiments, providing reference for the study of flame characteristics, thermophysical properties, and “safe distance-time” of tank fires of the same type.

## 2 Design of experiments

### 2.1 Engineering background

**Fig 1:**
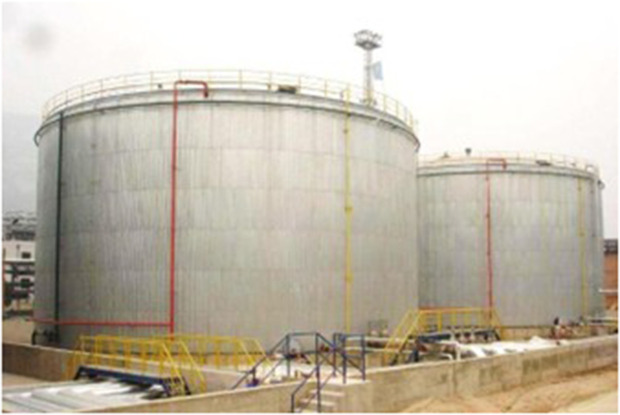
Field conditions.

Large-capacity storage tanks are generally vertical cylindrical steel tanks. Depending on their geometric shapes and structural characteristics, these tanks are categorized into two main types: vault tanks and floating roof tanks. Vault tanks are further divided into self-supporting vault tanks and supported vault tanks. Similarly, floating roof tanks consist of two types: an external floating roof tank, and an internal floating roof tank. The oil storage tank of this project adopts a vertical cylindrical, self-supporting floating roof tank structure, with a diameter of 22 m, a total height of 14.27 m, and an effective volume of about 5000 m^3^, as shown in Fig 1.

To facilitate the calculation process, the diameter of the actual oil tank is simplified to 20 m and the height of the oil tank is 10 m.

### 2.2 Design principle

In the study of oil tank fires, a common assumption is that the combustion flame acts as a singular heat source. Factors such as thermal diffusion, viscous dissipation, and the impact of pressure on the flue gas flow during the combustion process are typically disregarded [26,27]. Therefore, the differential equation of flue gas flow control is simplified to obtain:

Continuous equation:


$$\frac{\partial \rho }{\partial t}+\frac{\partial \left(\rho u_{j}\right)}{\partial x_{j}}=0$$ (1)

Motion equation:


$$\frac{\partial \left(\rho u_{i}\right)}{\partial t}+\frac{\partial \left(\rho u_{j}u_{i}\right)}{\partial x_{j}}=-\frac{\partial p}{\partial x_{i}}+\left(\rho -\rho_{0}\right)g_{i}+\frac{\partial }{\partial x_{j}}\mu \left(\frac{\partial u_{i}}{\partial x_{j}}+\frac{\partial u_{j}}{\partial x_{i}}\right)+\frac{\text{1}}{\text{3}}\frac{\partial }{\partial x_{i}}\left(\mu \frac{\partial u_{j}}{\partial x_{j}}\right)$$ (2)

Energy equation:


$$\frac{\partial }{\partial t}\left(\rho c_{p}T\right)+\frac{\partial }{\partial x_{i}}\left(\rho u_{i}c_{p}T\right)=\frac{\partial }{\partial x_{i}}\left(\lambda \frac{\partial T}{\partial x_{i}}\right)+q$$ (3)

Concentration equation:


$$\frac{\partial \left(\rho C_{s}\right)}{\partial t}+\frac{\partial \left(\rho u_{i}C_{s}\right)}{\partial x_{i}}=\frac{\partial }{\partial x_{i}}\left(\rho D_{s}\frac{\partial C_{s}}{\partial x_{i}}\right)+m_{s}$$ (4)

State equation:


p=ρRT (5)

Heat conduction equation:


$$\left(\frac{\rho c}{\lambda }\right)\frac{\partial T_{s}}{\partial t}=\frac{\partial^{2}T_{s}}{\partial x_{s}^{2}}$$ (6)

Boundary conditions:


$$x_{s}=0,\lambda_{s}\frac{\partial T_{s}}{\partial x_{s}}=\frac{0.036\lambda Pr^{1/3}}{L}Re^{0.8}\left(T-T_{s}\right)$$ (7)

In the formula, t is time, s; u is the flue gas flow velocity, m/s; p is the pressure, Pa; g is the acceleration of gravity, m/s^2^; T is the flue gas temperature, K; C_p_ is the specific heat capacity of flue gas at constant pressure, J/(kg•K); ρ is the density, kg/m^3^; ρ_0_ is the ambient air density; R is the gas constant, 8.314J/(mol•K); q is the heat of combustion per unit volume of fire source, J/m^3^; C_s_ is the flue gas concentration, kg/m^3^; m_s_ is smoke output per unit volume of fire source, kg; D_s_ is the mass diffusion coefficient of flue gas; λ_s_ is the thermal conductivity of the wall. α is the convective heat transfer coefficient; s is the wall parameter; P_r_ is Prandtl number; Re is the Reynolds number.

The dimensionless array is obtained by dimensionless and normalized processing of the above control equations. According to the similarity criterion, the similarity relationship R_I_ of the characteristic parameters of the small-scale oil tank fire experiment is established, including geometric similarity R_x_, time similarity R_t_, temperature similarity R_T_, fire intensity similarity R_Q_, speed similarity (experimental environment similarity) R_v_.

In summary, R_I_ can be expressed as:


$$\begin{array}{c} {}*20cx_{m}=x_{n}\lambda_{L}\\ t_{m}=t_{n}{\lambda_{L}}^{0.5}\\ T_{m}=T_{n}\\ Q_{m}=Q_{n}{\lambda_{L}}^{2.5}\\ v_{m}=v_{n}{\lambda_{L}}^{0.5} \end{array}$$ (8)

In the formula, x_m_ is the model size, m; xn is the prototype size, m; λ_L_ is the proportional constant of length; t_m_ is the model time scale, s; t_n_ is the prototype time scale, s; T_m_ is the model temperature, °C; T_n_ is the prototype temperature, °C; Q_m_ is the model fire source intensity, w; Q_n_ is the prototype fire source intensity, w; v_m_ is the model ambient wind speed, m/s; v_n_ is the prototype ambient wind speed, m/s.

The Reynolds number (Re) and the Froude number (Fr) are important parameters to characterizing the heat and mass transfer in the process of oil tank fire. However, under the same fluid medium conditions, Re and Fr may fail to satisfy similar requirements concurrently. Therefore, when Re is large enough, the flow is in the second self-modeling area of Re, and at this time, it is not necessary to consider that the Re of the prototype is the same as that of the model. Re > 10^4^ is required, and the flow is in a turbulent state. Therefore, for the model:


$$\frac{v_{m}L_{m}}{\upsilon_{m}}>10^{4}$$ (9)

In the formula, L_m_ is the equivalent diameter of the tank model; the viscosity of air at 50 °C is 1.86 × 10^-5^m^2^/s.

### 2.3 Experimental scheme

According to Section 1.1, the equivalent diameter of the oil tank is 20 m, and the height of the oil tank is 10 m. The equivalent diameter of the model in the similar experiment is 0.2 m, and the height of the model is 0.1 m. In terms of fuel selection for experiments, methanol-gasoline blends produce less black smoke during combustion, which aids in reducing the deviation of radiant heat flux caused by black smoke [28]. This facilitates the establishment and verification of the proportional relationship between small-scale pool fires and large-scale tank fires. Furthermore, the combustion process of methanol-gasoline blends is relatively stable, and the response of their flame characteristics and thermophysical properties to variations in ambient wind speed may be more predictable and manageable [29]. Additionally, methanol is a fuel with a relatively high oxygen content, and its combustion products are relatively clean, generating fewer harmful emissions [30]. This aligns with the current focus on environmental protection and sustainability. Therefore, methanol gasoline was used as the fuel for this experiment.

λ_L_=0.01

v_m_ > 0.93m/s

According to the experimental design, the wind speed for this experiment was set with a range of 1–4 m/s in 0.5 m/s intervals, resulting in a total of 7 experimental sets. It should be noted that this wind speed range was determined based on prior research and is expected to be an appropriate range for achieving the experiment’s objectives [14]. It is expected that the results from these experiments will be valuable in the context of the broader scientific community.

The present experiment utilized mechanical ventilation, ensuring the experimental environment remained unaffected by external wind fields. The experimental setup primarily comprised of testing devices, data acquisition systems, and wind-speed control systems.

**Fig 2:**
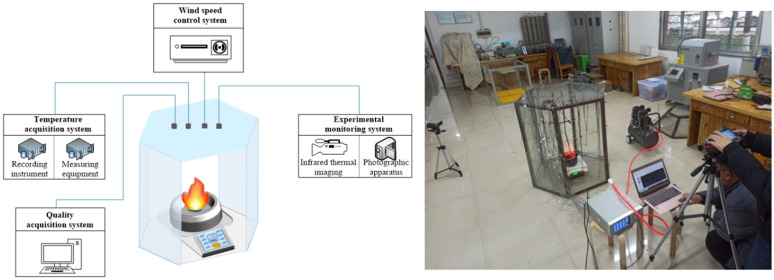
Experimental system structure.

To better monitor the temperature changes in all directions of the oil pool fire, a regular hexagonal prism model cabinet used within the test device. Each side of the model cabinet’s base measures 0.5m, with the prism extending a length of 1m. The model cabinet is constructed with an open structure on its two parallel sides and the top, facilitating ventilation from the system during the experiment. Such an arrangement allows for sufficient oxygenation during fuel combustion and permits the placement of additional system equipment within the model cabinet. The testing apparatus includes a stainless steel oil tank with a diameter of 0.2m. The data acquisition system mainly includes two parts: a temperature acquisition system and an experimental monitoring system. The former is composed of 32 K-type (nickel-chromium-nickel-silicon) armored thermocouples, MT-32X 32-channel temperature recorders, and Temp temperature recording software, designed to be compatible with the temperature recorder. The experimental monitoring system uses the FLIR non-cooled handheld infrared thermal imager to record the temperature field distribution of the oil pool fire. The wind speed control system consists of three parts: an air compressor, an air duct and a wind speed detector. The air compressor generates compressed air that, after passing through the closed ventilation duct system, forms a uniform airflow at a specific speed. The wind speed detector is employed to adjust the wind speed to meet experimental requirements. The structure of the experimental system is shown in Fig 2.

**Fig 3:**
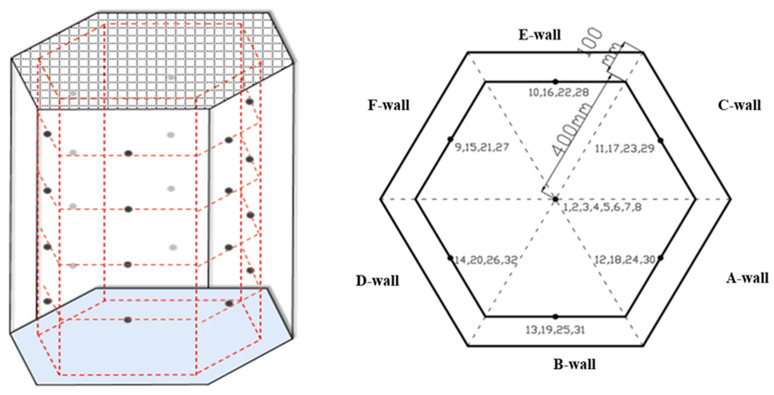
Temperature measuring point layout diagram.

To measure the combustion temperature-related parameters of methanol gasoline at various wind speeds. This experiment was carried out in the fire laboratory of Hunan University of Science and Technology. The experimental duration ranged from January to February, in which the temperature varied between 1°C −15°C, external air humidity ranged between 50% −80%, and the air pressure was maintained at standard atmospheric pressure. To thoroughly scrutinize the variations in combustion temperature, 32 temperature measuring points were designated in the configuration. In the monitoring of the temperature of the flame axis, it is considered that the temperature on the flame axis decreases obviously with the increase of the axis. With the increase of wind speed, the temperature induced by different heights of the axis will be greatly different. Consequently, a temperature measuring point (THCP) was incorporated every 0.1 m above the flame, resulting in a total of eight such points (THCP1-THCP8) along the flame axis. The model cabinet’s wall simulation included a cumulative four layers, aggregating 24 measuring points, arranged vertically every 0.1 m. The top layer contained six points (THCP9-THCP14); every subsequent layer followed at 0.2 m intervals, placed 0.8 m, 0.6 m, 0.4 m, and 0.2 m from the base of the model cabinet for the second (THCP15-THCP20), third (THCP21-THCP26), and fourth (THCP27-THCP32) layers, respectively. It is noteworthy that THCP12, THCP18, THCP24, and THCP30 were positioned on the wind speed inlet wall while THCP9, THCP15, THCP21, and THCP27 were strategically placed on the wind speed outlet wall. Fig 3 provides a schematic layout of the arrangement of temperature measuring points. These observational points should facilitate a comprehensive evaluation of the variations and behavior of flame temperatures at different heights and wind speeds.

The quality acquisition system is primarily designed for acquiring data on the quality of the oil inside the tank during combustion. The quality acquisition system principally consists of three components: an electronic scale, a timer, and aluminum silicate cotton above it. During the experiment, an alumina silicate wool is employed on the scale pan to thermally isolate the oil tank from the electronic scale. Subsequently, the timer is initiated, and the mass loss was recorded every 5s, with three repetitive measurements taken at each time point with an interval of ±0.5s before and after the designated time. The final result was obtained by averaging the three recordings. This collected information enables the calculation of the rate of combustion intensity, contributing crucial insights into the burning strength of the oil product.

## 3 Analysis of results

### 3.1 Combustion process analysis

**Fig 4:**
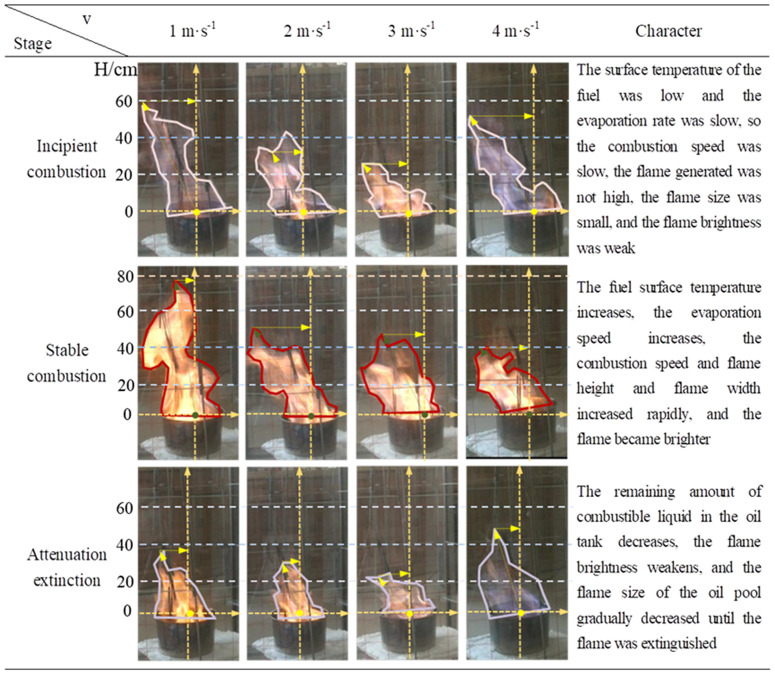
Methanol gasoline combustion process.

In order to depict the flame characteristics of the combustion process, the combustion of methanol gasoline can be categorized into three distinct steps. An illustrative example is presented herein, focusing on the flame state analysis under varying wind speeds of 1m/s, 2m/s, 3m/s, and 4m/s. The experimental findings were summarized in Fig 4.

From Fig 4, it can be observed that the flame of methanol gasoline underwent a notable color transition from light blue to red and back to light blue throughout the combustion process, particularly evident at a wind speed of 4m/s. Regarding the flame size, as the wind speed gradually increased, the height of the flame initially rose and subsequently decreased. However, the flame angle remained relatively unaffected during the combustion process. It should be noted that the aforementioned investigations lack a quantitative analysis of the combustion process in the context of oil tank fires, which will be further explored in sections 3.2 and 3.3.

### 3.2 Flame shape analysis

In order to conduct a quantitative analysis of the flame morphology during the combustion process, the flame image of methanol gasoline in a stable combustion state was BNN(Binary Neural Network) using Matlab. The resulting analysis highlighted the variations in flame height and flame inclination angle. Furthermore, Orloff’s method was employed to estimate the surface area and volume of the turbulent flame. This investigation aimed to elucidate the impact of wind speed on the flame morphology [31].

#### 3.2.1 Flame height.

**Fig 5:**
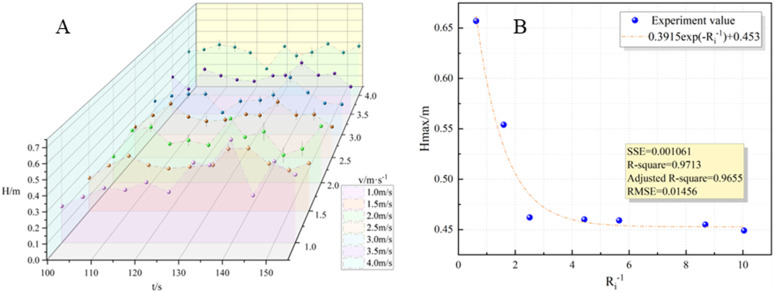
Flame height variation characteristics. **(A)** The relationship between flame height and time; **(B)** The relationship between flame height and Richardson number variation.


The intermittently fluctuation of flame height during the combustion process can be observed in Fig 5(A), which was a common pulsation phenomenon in flame combustion. Moreover, the influence of external wind on the behavior of methanol gasoline flame combustion and its interaction with the surrounding air needed to be examined. To gain a better understanding of the relative intensity between the thermal buoyancy effect of the oil pool fire and the speed of the external wind, the maximum flame height value was taken at each wind speed. The Richardson number were used for representation [32]. This parameter represents the ratio of the buoyancy growth rate of the combustion product to the transverse momentum generated by the external wind acting on the combustion product. When the oil pool fire is in a state of stable combustion, the Richardson number is given as follows:

$$R_{i}=\frac{\Delta \rho }{\rho_{a}}\frac{gL_{m}}{v^{2}}=\left(1-\frac{T_{a}}{T_{f}}\right)\frac{gL_{m}}{v^{2}}$$ (10)

In the equation, Δρ is the difference between the density of the air around the combustion product and the density of the fuel product, ρ_a_ is the transverse airflow density, g is the gravity acceleration, v is the wind speed, T_a_ is the transverse gas temperature, T_f_ is the adiabatic flame temperature, here values are 280 K and 1000 K, respectively.

Because the v is 0m/s in the absence of wind, to ensure the meaningfulness of the Richardson number (R_i_) under windless conditions, we adopted the reciprocal value (R_i_^-1^) for analysis. Fig 5(B) illustrates the maximum flame height (H_max_), and the fitting results indicate the following findings:


$$H_{\max}=0.3915e^{-{R_{i}}^{-1}}+0.4528$$ (11)

When the wind speed varied from 1 m/s to 4 m/s, the reduction rate of flame height displayed a gradual decrease. As the wind speed exceeded 2 m/s, corresponding to a reciprocal Richardson number (R_i_^-1^) exceeding 2.8, the flame height no longer decreased with increasing R_i_^-1^ values. Instead, it converged to a steady value of 0.4528m. These observations implied that in practical tank fire scenarios, a continuous increase in external wind speeds did not significantly alter the flame height.

#### 3.2.2 Flame inclination.

Based on the definition provided by SFPE, flame inclination under transverse wind conditions refers to the deviation of the flame’s center angle from the vertical direction. Previous studies, such as those referenced by [33,34], had investigated the relationship between flame inclination and wind speed.

**Fig 6:**
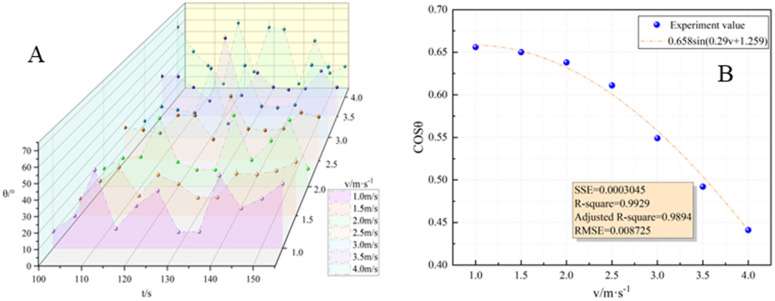
Variation characteristics of flame inclination. **(A)** Relationship between flame Angle and time variation; **(B)** The relationship between Angle cosine and wind speed.

From Fig 6(A), flame inclination can vary within a range of 8° to 65° when the wind speed at the fire source section ranged from 1 m/s to 4 m/s. The maximum flame inclination increased from 48.9° to 64.1° as the wind speed increased. It is important to note that under windless conditions, the flame exhibited a natural combustion state with a perpendicular shape relative to the oil pool surface.

When exposed to external wind, the transverse momentum exerted on the flame surpassed the buoyancy generated by its own combustion. As a result, the flame tilted, and the degree of tilt was governed by the strength of the external wind. To characterize the relationship between flame inclination angle and wind speed, the maximum inclination angle at each wind speed was taken. This functional equation was then fitted with the flame inclination angle formula proposed by Thomas. Referring to Thomas’ flame inclination formula, it is fitted to give:


Cos\nolimitsθ=0.658sin(0.277v+1.3) (12)

The fitting results indicate that at a wind speed of 6.64 m/s, the maximum inclination angle of the flame reached approximately 90°. Conversely, at a wind speed of 0.97 m/s, the maximum inclination angle was around 49°. It is worth noting that for wind speeds below 0.97 m/s, an unexpected trend is observed: as the wind speed increases, the flame inclination decreases, the experimental results are shown in Fig 6(B). The reliability of the fitted function is low in this case. Moreover, according to the wind speed requirements outlined in Section 1.3, the minimum prescribed wind speed is 0.93 m/s.

In summary, flame inclination under transverse wind conditions is influenced by the wind speed, with higher wind speeds resulting in greater flame tilting. The findings highlight the importance of external wind factors in determining the behavior of oil tank fires. The flame inclination angle formula proposed by Thomas can be used to analyze and predict the changing trend of flame inclination in response to varying wind speeds. However, the function does not accurately reflect the behavior of the flame at wind speeds below 0.97 m/s. Thus, more comprehensive analysis and investigation are needed to improve the reliability and applicability of the fitting function in these cases.

### 3.2.3 Flame surface area

**Fig 7:**
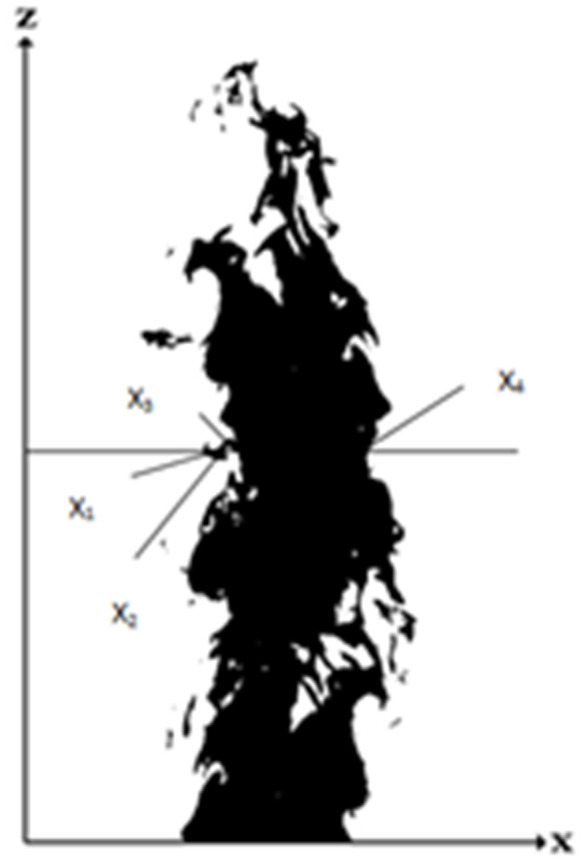
Flame diagram.

As shown in Fig 7 the black part is the flame area. According to the cylinder method, the flame diameter d($z_{i}$) of the image at $z_{i}$ height was calculated. The flame of the image at $z_{i}$ height was composed of two small cylinders with diameters of $x_{2}-x_{1}$ and $x_{4}-x_{3}$, respectively.

The equivalent diameter of the flame at $z_{i}$ height was:


$$d(z_{i})=[\sum_{j=1}^{m}{\left(x_{2j}-x_{2j-1}\right)}^{2}{]}^{1/2}$$ (13)

In the formula, m denotes that the height flame is composed of m cylinders, and i denotes the i-th layer of the flame.

The flame surface area S was obtained, and the calculation formula is as follows:


$$\Delta S_{i}=\pi \sum_{j=1}^{m}\left(x2j-x_{2j-1}\right)\Delta z$$ (14)

**Fig 8:**
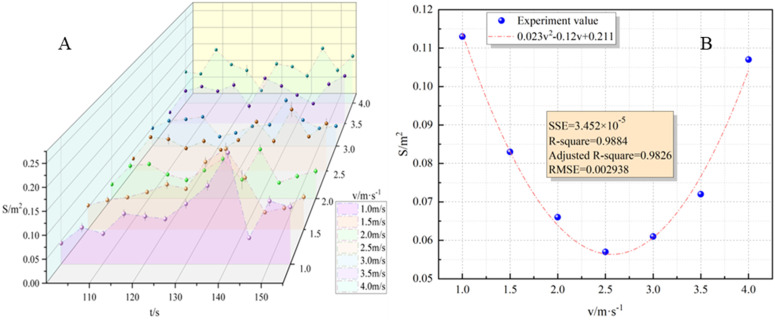
Variation characteristics of flame surface area. **(A)** Relationship between flame surface area and time; **(B)** Relationship between flame surface area and wind speed).


The analysis of Fig 8 reveals that there was no significant fluctuation in the flame surface area with respect to time under different wind speeds. Particularly at higher wind speeds, the difference between the maximum and minimum flame surface areas was negligible. Interestingly, the flame surface area initially decreased and then increased as the wind speed increased. To establish a more quantitative understanding of the relationship between wind speed and flame surface area, the average flame surface area was selected for further characterization. The obtained fitting results were presented below:

$$\overset{―}{S}=0.023v^{2}-0.12v+0.211$$ (15)

The fitting formula was solved and it was concluded that when the wind speed was 2.587m/s, $\overset{―}{S}$ was 0.055m^2^.

#### 3.2.4 Flame volume.

The flame volume V can be obtained by a similar method:


$$\Delta V_{i}=\frac{\pi }{\text{4}}d^{2}(z_{i})\Delta z$$ (16)

$$V=\sum_{j=1}^{n}\Delta V_{j}$$ (17)

In the formula, Δz denotes the height of the first flame cylinder.

**Fig 9:**
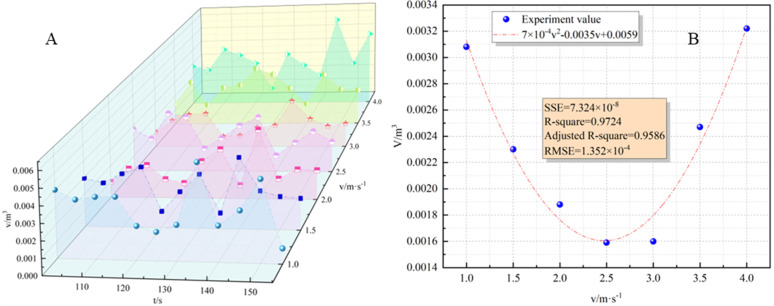
Flame volume variation characteristics. **(A)** Relationship between flame volume and time; **(B)** Relationship between flame volume and wind speed).


Fig 9 shows that with the increase of wind speed, the flame surface area first decreased and then increased, which was the same as the changing trend of flame surface area. To further clarify the relationship between wind speed and flame surface area, the average flame volume was selected for further quantitative characterization. The fitting results were as follows:

$$\overset{―}{V}=7\times 10^{-4}v^{2}-0.0035v+0.0059$$ (18)

The fitting formula was solved: when the wind speed is 2.482 m/s, it is 0.00155m^3^.

From the analysis of the average flame surface area and the average flame volume, it can be seen that the changing trend of the two was highly similar. Further explore the relationship between the two and fit it:


$$\overset{―}{V}=0.0285\overset{―}{S}(R^{2}=0.9088)$$ (19)

### 3.3 Thermophysical properties analysis

#### 3.3.1 Temperature variation characteristics.

**Fig 10:**
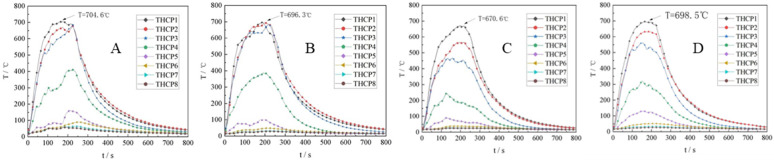
Flame axis temperature change. (A) v = 1m/s; (B) v = 2m/s; (C) v = 3m/s; (D) v = 4m/s.

Fig 10 clearly illustrates the temperature data recorded by thermocouples THCP1, THCP2, and THCP3, which displayed higher temperatures due to their proximity to the flame core and the location of the internal and external flame. On the other hand, the temperatures detected by THCP4 to THCP8 gradually decreased as the thermocouples were positioned at higher place.

When the wind speed fell within the range of 1 m/s to 3 m/s, the flame temperature exhibited a decreasing tendency. This was attributed to the fact that the external airflow carries away a portion of the flame’s heat, resulting in a decrease in temperature (T) as the wind speed (v) increased.

However, at wind speeds exceeding 3 m/s, there was a notable upward trend in the detected flame temperature. This was due to the stretching of the flame caused by lower wind speeds, which promoted increased heat exchange between the flame and the fuel surface. Consequently, this accelerated heat exchange led to a higher combustion rate, resulting in an increase in the maximum flame temperature.

In summary, the temperature variations observed by the thermocouples indicate distinct patterns related to their proximity to the flame, elevation, and the influence of wind speed. These findings provide valuable insights into the thermal behavior of the flame in relation to different operating conditions and further our understanding of the combustion process.

To further investigate the temperature field changed around the flame, an analysis was conducted using the temperature data obtained from the four-layer thermocouples positioned on each wall of the hexagonal prism model cabinet. It was observed that the temperature measured by the thermocouples around the flame exhibited a certain degree of delay. Thus, the thermocouples positioned around the flame were used to calculate the average temperature within the time period of 150s ~ 250s.

In order to analyze the temperature changes on both sides of the airflow path, the average temperature ${\overset{―}{T}}_{A}$ recorded at the upwind side’s A-wall measuring point was subtracted from the average temperature ${\overset{―}{T}}_{F}$ at the downwind side’s F-wall measuring point. This allowed for the determination of temperature variations at different heights of the walls on both sides of the flame. Additionally, by subtracting the average temperature ${\overset{―}{T}}_{C}$ at the C-wall measuring point from the average temperature ${\overset{―}{T}}_{B}$ at the B-wall measuring point on the wind flow path, and likewise subtracting the average temperature ${\overset{―}{T}}_{E}$ at the E-wall measuring point from the average temperature ${\overset{―}{T}}_{D}$ at the D-wall measuring point on the opposite side, temperature changes on both sides of the wind flow path at different heights could be obtained.

**Fig 11:**
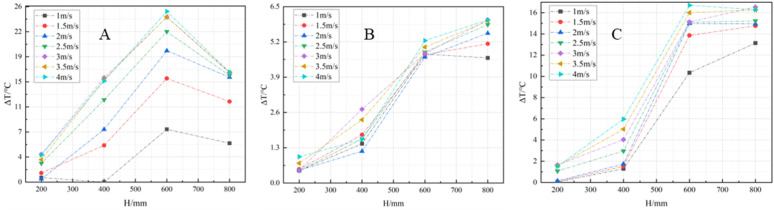
Temperature variation of flame axis. **(A)** ${\bar{T}}_{F}-{\bar{T}}_{A}$; **(B)** ${\bar{T}}_{B}-{\bar{T}}_{C}$; **(C)** ${\bar{T}}_{D}-{\bar{T}}_{E}$.

Analysis of Fig 11(A) reveals that the temperature of A-wall, located on the upwind side of the flame, is lower compared to the temperature of the F-wall on the downwind side of the flame. This temperature difference arose due to the behavior of the flame region when exposed to high wind speeds. Specifically, at higher wind speeds, the flame region experienced skewing and stretching along with the movement of the airflow field. And when wind speed increases, the deflection degree of the flame became larger. Consequently, the temperature recorded by the thermocouples on the downwind side’s wall F is higher than that recorded by the thermocouples on the upwind side’s wall A.

Furthermore, Fig 11(B) demonstrates that the maximum temperature difference detected under this specific range of wind speeds was 6.01°C. The average temperature difference gradually increased as the height of the surrounding monitoring points increased. Similarly, Fig 11(C) shows that the maximum temperature difference detected under this range of wind speeds was 16.54°C. Moreover, the temperature difference increases with the height arrangement of the surrounding monitoring points until it reached a certain value and then tended to flatten out.

Collectively, these observations highlight the impact of wind speed on the temperature distribution around the flame. The temperature differences between the upwind and downwind sides of the flame provide valuable insights into the heat transfer behavior and thermal characteristics in the vicinity of the flame, contributing to a deeper understanding of the complex dynamics involved in oil tank fires.

#### 3.3.2 Variation characteristics of heat release rate.

The heat release rate (HRR) is a critical parameter that reflects the speed and magnitude of heat release from a fire source, which also characterizes its ability to release heat. A greater HRR of the fire source results in a larger amount of heat being fed back to the surface of the material, which accelerates the rate of material pyrolysis and increases the amount of volatile combustible substances. This in turn expedites the propagation of flames.

In fire, HRR is generally calculated according to the following formula:


$$Q=\Phi \times \overset{\cdot }{m}\times \Delta H$$ (20)

According to relevant research, combustion efficiency is generally related to the size of the oil tank, the coefficient of the oil pool wall surface, and wind speed [35, 36] . In this study, the oil tank used remained unchanged, and the oil level was far below the height of the tank wall. Therefore, the external wind speed had a minimal impact on the combustion process within the tank. Based on this, in the formula, Ф is the combustion efficiency factor, taking 0.8; $\overset{\cdot }{m}$ is the mass burning rate of combustibles, kg/s; ΔH is the calorific value of combustibles, taking 26.78 MJ / kg.

**Fig 12:**
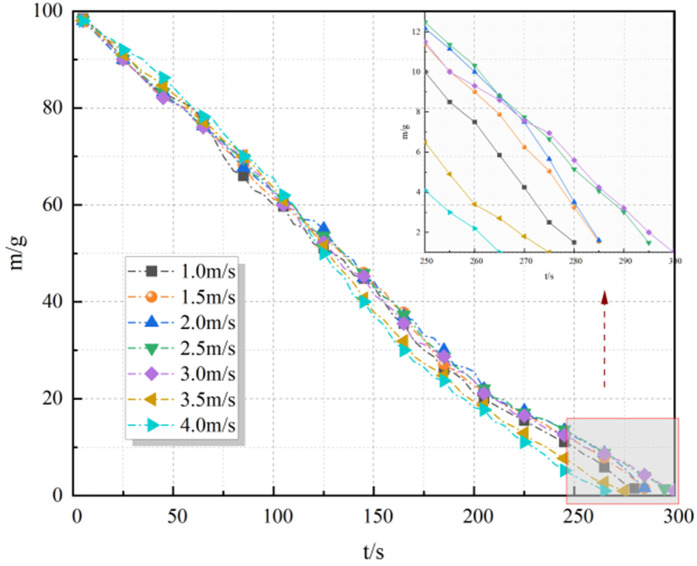
Fuel quality change rule.

It can be seen from Fig 12 that the fuel mass changes approximately linearly with time, so $\overset{\cdot }{m}$ was a fixed value, and the results of $\overset{\cdot }{m}$ and Q at different wind speeds are shown in Table 1.

**Table 1:** Heat release rate variation characteristics.

v/m·s-1	1	1.5	2	2.5	3	3.5	4
$\overset{\cdot }{m}$/g	0.352	0.345	0.345	0.334	0.33	0.36	0.374
Q/kw	7.541	7.391	7.391	7.156	7.07	7.713	8.013

The experimental data in Table 1 show that m increased from 0.33 g/s (3 m/s) to 0.374 g/s (4 m/s), contributing to a 13.3% rise in HRR. This acceleration is attributed to enhanced convective heat transfer at higher wind speeds, which promotes fuel vaporization [37,12].

Combustion efficiency Φ (assumed constant at 0.8 in Section 3.3.2) may also increase due to improved air entrainment under strong wind conditions. Studies suggest Φ can rise by 5–10% when wind speed exceeds 3 m/s, as turbulence enhances fuel-air mixing [38]. Assuming Φ increases to 0.85 at 4 m/s, the combined effect of m and Φ amplifies HRR by 20.2% (vs. 13.3% from m alone), aligning with the observed jump from 7.07 kW to 8.01 kW.

## 4 Discussion of results

### 4.1 Result validation

To verify the reliability of the experimental results, a tank model was established using Pyrosim based on the tank model in Section 1.1 with an equal proportion scale. Eight thermocouples were arranged at intervals of 10m along the centerline of the flame. According to the similarity relationship of R_v_ and the Reynolds number similarity principle, four sets of simulation experiments were designed.

Since meshing is crucial to the running results and running time, According to the Pyrosim manual, when 1/16 ≤ d/D ≤ 1/4, higher computational accuracy can be achieved [39]. And a large number of studies have proved that when d is less than or equal to 0.1D, it is possible to ensure higher accuracy of the simulation results [40]. where D is the characteristic diameter of the fire source.


$$D=(\frac{Q}{\rho_{a}c_{p}T_{a}g^{\frac{\text{1}}{\text{2}}}}{)}^{\frac{\text{2}}{\text{5}}}$$ (22)

In the formula, Q is the power of the fire source, and 8.1 × 10^5^kw is taken according to the similarity principle and the previous experimental results. ρa is air density, 1.29 kg/m^3^; c_p_ is the specific heat capacity of air, 1kJ/(kg·K); T_a_ is the ambient temperature, 288K; g is the gravitational acceleration, 9.8m/s^2^.

Therefore, D = 13.71m, d ≤ 1.371m, and the design mesh diameter was 1m × 1m × 1m. During the simulation process, the Large Eddy Simulation (LES) method was employed to address turbulence. A suitable LES model for tank fire scenarios was selected, and relevant parameters were set. For potential SGS (Sub-Grid Scale) turbulence, its impact was indirectly considered by adjusting the turbulence viscosity coefficient function in the model.

The Smagorinsky-Lilly subgrid-scale (SGS) model was adopted to resolve turbulent motions at unresolved scales. This model is widely used in fire simulations due to its simplicity and effectiveness in capturing buoyancy-driven flows and flame-turbulence interactions [41, 38]. The Smagorinsky constant Cs was set to 0.18, a value validated in previous studies on pool fires and enclosure fires [42,43]. To enhance adaptability to local flow conditions, the dynamic procedure was applied to adjust Cs based on resolved-scale strain rates, ensuring energy consistency between resolved and subgrid scales [44]. Additionally, the turbulent Prandtl number (Pr_t_) and turbulent Schmidt number (Sc_t_) were set to 0.7 and 0.4, respectively, aligning with recommendations for combustion simulations [45].

**Fig 13:**
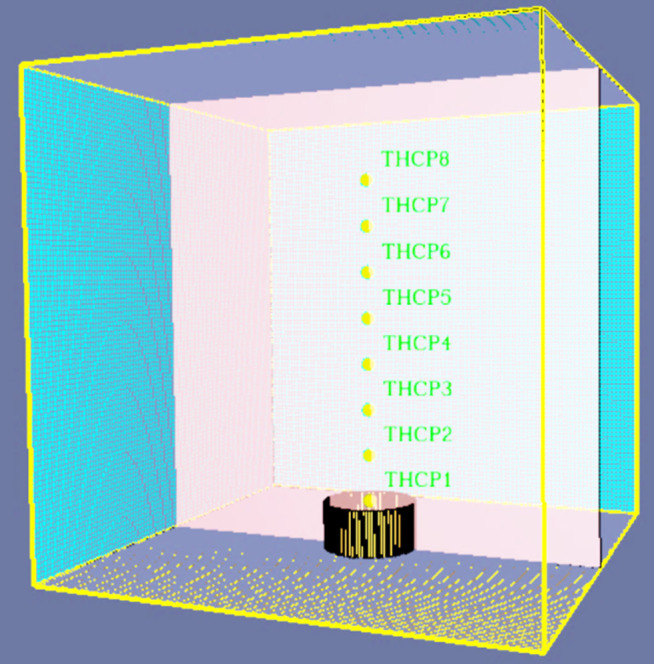
Physical models and meshing.

**Fig 14:**
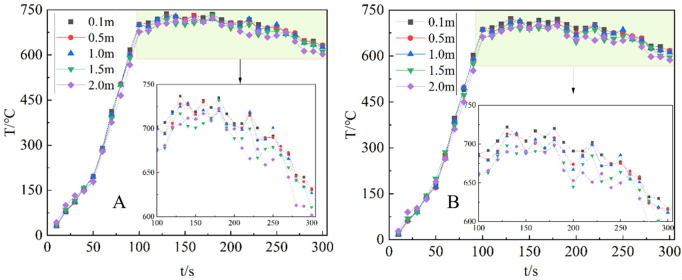
Grid independence verification of v = 0m/s. **(A)** THCP1; **(B)** THCP 8.

Besides, a detailed chemical reaction mechanism and radiation model were added to consider these interactions. The physical model is shown in Fig 13. And the results of grid independence verification are shown in detail in Fig 14.

**Fig 15:**
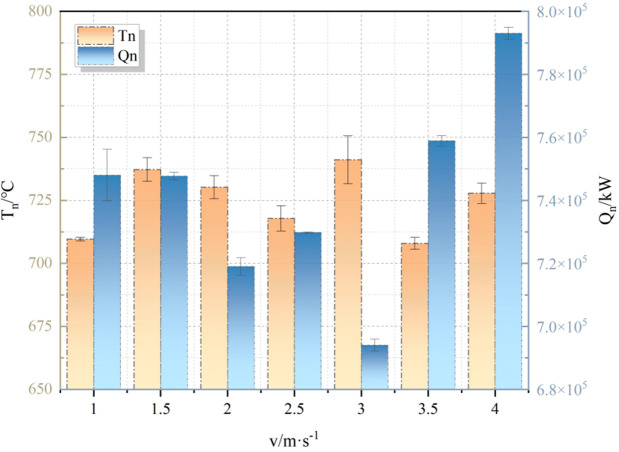
Result verification.

The accuracy of the simulation experiment was verified by using the temperature and HRR monitoring results of numerical simulation, and according to the similarity relationship R_T_ and R_Q_. The results are shown in Fig 15.

It can be seen from Fig 15 that the temperature of the simulated experiments was approximately 40°C higher than the theoretical values of similar experiments, with an average E_Tm_ error of 4.41%. Additionally, as shown in Fig 15, when v ≤ 2.5 m/s, there was little difference in Q_n_, indicating that a low wind speed will not affect HRR. The average error E_Qm_ of the experiment was 2.54%, both of which met the experimental error requirements and verified the accuracy of the analogous experiment.

### 4.2 Security analysis

The above research had obtained the characteristics of flame and thermophysical properties of tank fires. To further clarify the damage caused by thermal radiation to human skin, this section studied it. In the air, the propagation of thermal radiation can be expressed as [46,47]:


$$q=\frac{\zeta Q}{4\pi R^{2}}$$ (23)

In the formula, Q is the heat release rate of fire source, kw; ζ is the radiation coefficient, take 1/3; r is the distance from the flame center, m; q is thermal radiation, kw/ m^2^.

According to C. M. Pietersen ‘s research [48], the degree of damage to the human caused by thermal radiation can be expressed as:


$$P_{r}=\left\{ \begin{array}{c} -39.83+3.0186q^{\frac{\text{4}}{\text{3}}}\ln t(First-degree\;burn\;curve)\\ -43.14+3.0188q^{\frac{\text{4}}{\text{3}}}\ln t(Second-degree\;burn\;curve)\\ -37.23+2.56q^{\frac{\text{4}}{\text{3}}}\ln t(Thermal\;death\;time\;curve) \end{array}\right.$$ (24)

According to the similarity relationship R_t_, R_x_, R_Q_, the degree of thermal radiation damage to the human when the tank fire occurs can be expressed as:


$$P_{r}=\left\{ \begin{array}{c} -39.83+\frac{2.391{Q_{n}}^{\frac{\text{8}}{15}}}{{R_{n}}^{2}}\ln\frac{t_{1}}{10}(First-degree\;burn\;curve)\\ -43.14+\frac{2.391{Q_{n}}^{\frac{\text{8}}{15}}}{{R_{n}}^{2}}\ln\frac{t_{2}}{10}(Sec\mathrm{\backslash nolimits}ond-degree\;burn\;curve)\\ -37.23+\frac{2.028{Q_{n}}^{\frac{\text{8}}{15}}}{{R_{n}}^{2}}\ln\frac{t_{3}}{10}(Thermal\;death\;time\;curve) \end{array}\right.$$ (25)

**Fig 16:**
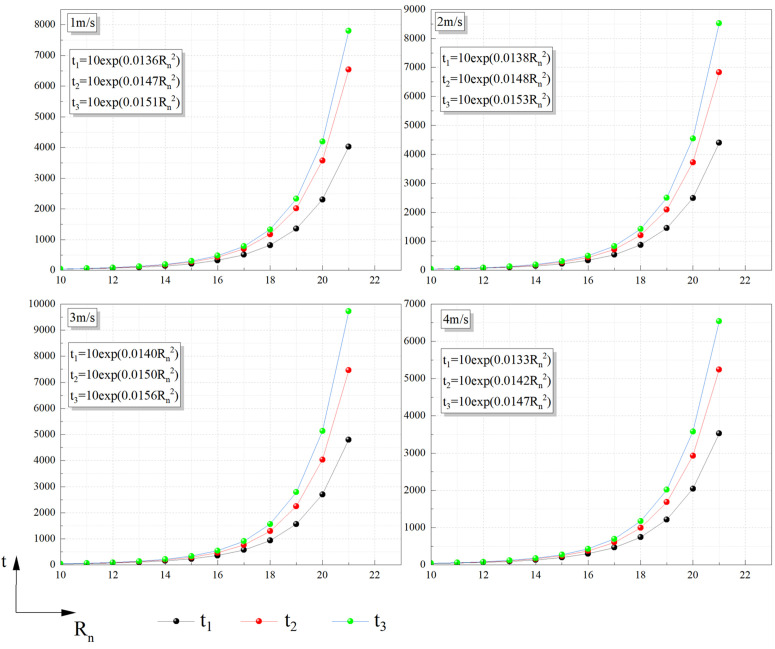
“Safe distance-time” critical curve.

Previous studies have determined that the critical value of P_r_ is 5 [15], and Q_n_ under different wind speeds was substituted to determine its “safe distance-time” critical curve as shown in Fig 16 (Note: When studying the influence of v on HRR, it has been found that the change of HRR was not obvious when the variation of wind speed was small, especially when v ≤ 2.5 m/s. Therefore, only 1 m/s, 2 m/s, 3 m/s, and 4 m/s injury models were considered in the study of thermal radiation injury).

Fig 16 depicts an exponential growth relationship between the time and distance required for thermal radiation to cause damage. When a person was located 10 to 16m away from the flame center, the time required for thermal radiation to cause damage was not significantly different, and the time required for death and injury was also relatively short. Taking the combustion duration of 3000s as an example, the safe distance between it and the flame center was at least 21m. When it exceeded 21m, the time required for thermal radiation to cause damage increased sharply, and the increase in the time required for thermal radiation to kill was even more significant.

### 4.3 Discussion

In this paper, focusing on the flame shape and thermal physics attributes around the environment wind, the flame characteristics of the height, inclination angel, surface area and volume, and the flame temperature and heat release rate, the theory is extended and practical guidance is obtained on the fire safe. Besides, based on the extensively wind velocity range designed by similarity principle, qualitative research on the flame morphology [49,50] and deepened response model by the experimental data. At the same time, assuming that the model is conducted under flat terrain conditions without dynamic movement of personnel, and using clean fuel (methanol gasoline) to reduce smoke interference, there is an entirely new sight in knowledge of flame on fire. Compared with the existing experiments, this is an essential progress. On the other hand, different from the past research on the mechanism [51] and model of heat transfer [52], the study focuses on the effects of thermal radiation on personnel safety and the constructed fire safety distance model has practical guiding significance for fire risk assessment and emergency response.

Although this study has made significant theoretical and practical contributions, several areas warrant further investigation. Notably, the effects of tank fires under more complex conditions—such as varying weather patterns and changes in oil properties—have not been adequately addressed. Understanding how these factors influence flame morphology and heat release rates requires additional research. Furthermore, this study primarily examines a single tank fire, whereas real-world scenarios may involve the interaction of multiple tanks. In such instances, the dynamics of flame propagation, thermal radiation, and convection are likely to be more intricate. Future research should build upon these findings by incorporating greater experimental complexity, including the introduction of diverse environmental factors and oil characteristics, to more accurately simulate actual fire conditions. Additionally, exploring more sophisticated fire models that account for interactions between oil tanks and fire spread in various building structures will enhance theoretical support for fire risk assessment and emergency response strategies.

## 5 Conclusion

(1) The similarity experiment of tank fire was conducted to investigate the flame shape characteristics. The results showed that with the increase of wind speed, the influence of wind speed on flame height became smaller and smaller. By studying the flame inclination angle, it was found that the wind speed condition of the experiment needed to be between 0.97m/s and 6.64m/s. The variation characteristics of flame surface area and volume were basically the same. With the increase of wind speed, it decreased first and then increased. There was a linear relationship between flame volume and surface area.
(2) Based on the thermophysical properties of methanol gasoline, it was found that with the increase of wind speed, the flame temperature decreased first and then increased. This was because the external airflow took away part of the heat of the flame, and the flame temperature decreased with the increase of wind speed. When it reached a certain value and continued to increase, the flame was stretched lower by the wind speed, which accelerated the heat exchange between the flame and the fuel surface, also increased the combustion rate, and the flame temperature raised again. By studying heat release rate, it was found that the larger the flame volume, the higher the heat release rate, and there was a linear relationship between the two.
(3) Pyrosim was used for experimental verification. It was found that the temperature error and heat release rate error range of the simulation experiment were both less than 5%, which verified the reliability of similar experiments. By further studying the “safe distance-time” relationship of tank fires, it was found that the safe distance of personnel in this engineering background was at least 21 m.

## Nomenclature

**Table:** 

t	Time, (s)
u	Flue gas flow velocity, (m/s)
p	Pressure, (Pa)
g	Acceleration of gravity, (m/s^2^)
T	Flue gas temperature, (K)
$C_{p}$	Specific heat capacity of flue gas at constant pressure, (J/(kg•K))
ρ,$\rho_{0}$	ρ is the density; $\rho_{0}$ is the ambient air density, (kg/m^3^)
*R*	Gas constant, (8.314J/(mol•K))
q	Heat of combustion per unit volume of fire source, (J/m^3^)
$C_{s}$	Flue gas concentration, (kg/m^3^)
$m_{s}$	Smoke output per unit volume of fire source, (kg)
$D_{s}$	Mass diffusion coefficient of flue gas
$\lambda_{s}$	Thermal conductivity of the wall
α	Convective heat transfer coefficient
s	Wall parameter
$P_{r}$	Prandtl number
$R_{e}$	Reynolds number
*R*_*I*_	The similarity relationship of the characteristic parameters of the small-scale oil tank fire experiment
*R*_*x*_	Geometric similarity
*R*_*t*_	Time similarity
*R*_*T*_	Temperature similarity
*R*_*Q*_	Fire intensity similarity
*R*_*v*_	Speed similarity (experimental environment similarity)
$x_{m}$,$\;x_{n}$	$x_{m}$ is the model size; $x_{n}$ is the prototype size, (m)
*λ*_*L*_	Proportional constant of length
$t_{m}$,$\;t_{n}$	$t_{m}$ is the model time scale; $t_{n}$ is the prototype time scale, (s)
$T_{m}$,$\;T_{n}$	$T_{m}$ is the model temperature; $T_{n}$ is the prototype temperature, (°C)
$Q_{m}$,$\;Q_{n}$	$Q_{m}$ is the model fire source intensity; $Q_{n}$ is the prototype fire source intensity, (w)
$v_{m}$,$\;v_{n}$	$v_{m}$ is the model ambient wind speed; $v_{n}$ is the prototype ambient wind speed, (m/s)
$F_{r}$	Froude number
$L_{m}$	Equivalent diameter of the tank model, (m)
Δρ	The difference between the density of the air around the combustion product and the density of the fuel product
$\rho_{\alpha }$	Transverse airflow density, (kg/m^3^)
v	Wind speed, (m/s)
$T_{\alpha }$	Transverse gas temperature, (K)
$T_{f}$	Adiabatic flame temperature, (K)
*R*_*i*_	Richardson number
$H_{max}$	Maximum flame height, (m)
*d(*$Z_{i}$)	Flame diameter
*S*	Flame surface area, (m^2^)
$\bar{S}$	Average flame surface area, (m^2^)
*V*	Flame volume
Δz	The height of the first flame cylinder
$\bar{V}$	Average flame volume
${\bar{T}}_{A}$,$\;{\bar{T}}_{B}$,$\;{\bar{T}}_{C}$, ${\bar{T}}_{D}$, ${\bar{T}}_{E}$,${\bar{T}}_{F}$	Average temperature
Φ	Combustion efficiency factor
$\dot{m}$	The mass burning rate of combustibles, (kg/s)
ΔH	Calorific value of combustibles, (MJ/ kg)
*D*	The characteristic diameter of the fire source
*Q*	The power of the fire source, (w)
*ζ*	Radiation coefficient
*r*	The distance from the flame center, (m)
*q*	Thermal radiation, (kw/ m^2^)

## Supporting information

S1 Data Minimal data. (DOCX)
