# Supplementary material for: Research on similarity test design and characteristic verification for tank fires under environmental wind conditions
Source: PLoS One. 2026 Jan 9;21(1):e0340275. doi: 10.1371/journal.pone.0340275 (PMC12788686; doi:10.1371/journal.pone.0340275)
Supplement: S1 Data — (DOCX) [file pone.0340275.s001.docx]

**Fig.5 Flame height variation characteristics**

| t | 1m/s | error | 1.5m/s | error | 2m/s | error | 2.5m/s | error | 3m/s | error | 3.5m/s | error | 4m/s | error |
| --- | --- | --- | --- | --- | --- | --- | --- | --- | --- | --- | --- | --- | --- | --- |
| 99.82329 | 0.23489 | 0.005 | 0.22678 | 0.003 | 0.18992 | 0.01 | 0.23245 | 0.03 | 0.26982 | 0.01 | 0.31318 | 0.0065 | 0.3853 | 0.03 |
| 104.82523 | 0.29499 | 0.0043 | 0.31098 | 0.0063 | 0.37876 | 0.0065 | 0.32148 | 0.005 | 0.31163 | 0.005 | 0.21124 | 0.033 | 0.40422 | 0.02 |
| 109.79012 | 0.3517 | 0.012 | 0.37653 | 0.017 | 0.40264 | 0.033 | 0.40977 | 0.03 | 0.32557 | 0.042 | 0.37821 | 0.019 | 0.44802 | 0.042 |
| 114.79207 | 0.33996 | 0.0089 | 0.31323 | 0.009 | 0.28086 | 0.019 | 0.30987 | 0.02 | 0.32657 | 0.026 | 0.32416 | 0.023 | 0.42612 | 0.042 |
| 119.79401 | 0.38891 | 0.011 | 0.29018 | 0.014 | 0.30733 | 0.042 | 0.27632 | 0.042 | 0.17873 | 0.017 | 0.28919 | 0.017 | 0.37734 | 0.023 |
| 125.09237 | 0.32643 | 0.009 | 0.30129 | 0.01 | 0.2729 | 0.023 | 0.28731 | 0.026 | 0.26982 | 0.027 | 0.27689 | 0.017 | 0.23548 | 0.017 |
| 130.90945 | 0.51507 | 0.023 | 0.31381 | 0.023 | 0.46432 | 0.017 | 0.32942 | 0.017 | 0.27729 | 0.035 | 0.32377 | 0.017 | 0.43309 | 0.017 |
| 134.7628 | 0.49974 | 0.017 | 0.42391 | 0.027 | 0.33021 | 0.017 | 0.32129 | 0.027 | 0.3196 | 0.037 | 0.32671 | 0.027 | 0.34996 | 0.037 |
| 139.76474 | 0.66212 | 0.022 | 0.42849 | 0.024 | 0.37001 | 0.065 | 0.42421 | 0.035 | 0.45997 | 0.044 | 0.43315 | 0.035 | 0.38182 | 0.044 |
| 144.95194 | 0.30474 | 0.013 | 0.32382 | 0.025 | 0.20047 | 0.057 | 0.32414 | 0.037 | 0.33901 | 0.023 | 0.32811 | 0.014 | 0.45499 | 0.023 |
| 149.95389 | 0.52362 | 0.011 | 0.27893 | 0.023 | 0.24544 | 0.054 | 0.32813 | 0.044 | 0.2534 | 0.017 | 0.34271 | 0.01 | 0.37883 | 0.027 |
| 154.95583 | 0.43806 | 0.009 | 0.32647 | 0.017 | 0.4096 | 0.016 | 0.23429 | 0.014 | 0.24394 | 0.023 | 0.23136 | 0.023 | 0.43508 | 0.035 |

**Fig.6 Variation characteristics of flame inclination**

| t | 1m/s | error | 1.5m/s | error | 2m/s | error | 2.5m/s | error | 3m/s | error | 3.5m/s | error | 4m/s | error |
| --- | --- | --- | --- | --- | --- | --- | --- | --- | --- | --- | --- | --- | --- | --- |
| 99.91309 | 10.69156 | 0.5721 | 11.3628 | 0.5121 | 12.9041 | 1.223 | 24.7281 | 1.221 | 12.9041 | 1.198 | 32.9834 | 2.987 | 60.417 | 4.3998 |
| 105.08634 | 20.2225 | 1.109 | 23.1231 | 1.509 | 20.2225 | 1.7154 | 22.9831 | 2.089 | 24.137 | 1.721 | 32.6528 | 3.15 | 39.2844 | 2.87 |
| 109.9028 | 49.01391 | 1.98 | 32.2991 | 3.198 | 21.07348 | 1.6625 | 20.6381 | 1.523 | 21.58407 | 1.987 | 23.8345 | 1.354 | 27.20052 | 2.2315 |
| 115.00172 | 12.22332 | 1.21 | 13.2326 | 1.221 | 48.33312 | 1.509 | 33.8973 | 0.987 | 19.20133 | 1.915 | 12.7362 | 1.0509 | 11.54254 | 1.009 |
| 119.90738 | 26.51974 | 0.889 | 27.1838 | 1.989 | 17.49938 | 1.198 | 33.7456 | 1.915 | 54.11977 | 2.354 | 64.2367 | 3.598 | 63.82091 | 3.498 |
| 125.08062 | 36.05068 | 1.123 | 21.0912 | 1.23 | 11.88293 | 1.221 | 15.9821 | 2.354 | 10.86175 | 0.998 | 17.2639 | 1.1221 | 21.92446 | 1.221 |
| 130.07547 | 10.18097 | 0.7854 | 11.9213 | 0.7154 | 11.88293 | 0.987 | 47.9821 | 2.5721 | 56.33231 | 0.987 | 35.7362 | 2.4721 | 60.07661 | 4.987 |
| 135.07033 | 10.35117 | 0.6625 | 12.1259 | 0.6625 | 50.37547 | 1.15 | 23.7642 | 2.198 | 24.47739 | 2.415 | 23.9832 | 2.0198 | 58.37466 | 3.415 |
| 139.90166 | 45.58163 | 1.998 | 27.9824 | 2.998 | 18.86094 | 2.354 | 22.6531 | 1.987 | 23.28602 | 2.354 | 21.8328 | 1.5987 | 11.54254 | 0.6354 |
| 145.16409 | 24.98798 | 0.987 | 27.3919 | 1.987 | 32.47658 | 2.5721 | 23.9836 | 2.15 | 25.49856 | 1.687 | 22.7324 | 1.3998 | 48.50332 | 2.298 |
| 150.15895 | 31.11502 | 1.15 | 29.9381 | 2.15 | 46.97156 | 1.109 | 35.9278 | 2.354 | 56.6727 | 2.15 | 37.2653 | 1.87 | 20.73309 | 1.587 |
| 155.1538 | 40.47577 | 2.354 | 35.8213 | 3.354 | 12.7339 | 1.98 | 32.7289 | 2.054 | 55.31114 | 3.354 | 23.8548 | 2.3415 | 26.34954 | 1.3298 |

**Fig.8 Variation characteristics of flame surface area**

| 100.00229 | 0.04517 | 0.0025 | 0.05517 | 0.00288 | 0.0345 | 0.00208 | 0.03159 | 0.00603 | 0.04822 | 0.00201 | 0.03159 | 0.0031 | 0.09191 | 0.00317 |
| --- | --- | --- | --- | --- | --- | --- | --- | --- | --- | --- | --- | --- | --- | --- |
| 104.99714 | 0.07921 | 0.00978 | 0.06721 | 0.00878 | 0.07921 | 0.0087 | 0.08634 | 0.0046 | 0.06803 | 0.00677 | 0.09204 | 0.00397 | 0.08632 | 0.00332 |
| 110.08119 | 0.066 | 0.00643 | 0.07368 | 0.00343 | 0.08378 | 0.00643 | 0.08116 | 0.01341 | 0.0721 | 0.00443 | 0.10437 | 0.00286 | 0.15754 | 0.00231 |
| 115.09091 | 0.10868 | 0.0097 | 0.0857 | 0.0057 | 0.05838 | 0.0036 | 0.0578 | 0.00578 | 0.0787 | 0.00317 | 0.09133 | 0.00257 | 0.11376 | 0.00407 |
| 120.17496 | 0.10309 | 0.00992 | 0.10309 | 0.00994 | 0.04517 | 0.0076 | 0.07631 | 0.0081 | 0.02535 | 0.00332 | 0.10876 | 0.01112 | 0.09648 | 0.01595 |
| 124.99142 | 0.0975 | 0.00678 | 0.09315 | 0.00578 | 0.06854 | 0.00571 | 0.05397 | 0.00324 | 0.03552 | 0.00231 | 0.04943 | 0.01019 | 0.03704 | 0.0034 |
| 129.98628 | 0.13002 | 0.01012 | 0.1547 | 0.01112 | 0.09597 | 0.01012 | 0.07893 | 0.0061 | 0.0533 | 0.00407 | 0.12761 | 0.00201 | 0.11579 | 0.00812 |
| 135.15952 | 0.16922 | 0.01119 | 0.18446 | 0.01419 | 0.04618 | 0.02419 | 0.12571 | 0.00693 | 0.05736 | 0.00694 | 0.10608 | 0.00677 | 0.10766 | 0.0025 |
| 140.08005 | 0.24035 | 0.01324 | 0.11857 | 0.01024 | 0.11935 | 0.01224 | 0.07629 | 0.00908 | 0.1229 | 0.00801 | 0.08029 | 0.00443 | 0.08022 | 0.00978 |
| 145.16409 | 0.05685 | 0.0031 | 0.0393 | 0.0021 | 0.03856 | 0.0041 | 0.15704 | 0.01921 | 0.07006 | 0.00314 | 0.05626 | 0.00208 | 0.1616 | 0.00643 |
| 150.06975 | 0.13611 | 0.01132 | 0.04919 | 0.00732 | 0.05431 | 0.00632 | 0.08795 | 0.00578 | 0.04771 | 0.00341 | 0.11118 | 0.0087 | 0.09699 | 0.0097 |
| 155.06461 | 0.12341 | 0.01002 | 0.07448 | 0.00902 | 0.06651 | 0.00832 | 0.12138 | 0.01312 | 0.05381 | 0.00603 | 0.13436 | 0.00643 | 0.13865 | 0.0087 |

**Fig.9 Flame volume variation characteristics**

| t | 1m/s | 1.5m/s | 2m/s | 2.5m/s | 3m/s | 3.5m/s | 4m/s |
| --- | --- | --- | --- | --- | --- | --- | --- |
| 100.11086 | 0.00402 | 0.00295 | 9.76164E-4 | 0.00276 | 9.97469E-4 | 0.00148 | 0.00277 |
| 105.192 | 0.00347 | 0.00268 | 0.00185 | 0.00215 | 0.00179 | 0.00298 | 0.00247 |
| 110.184 | 0.00364 | 0.00325 | 0.00208 | 0.00381 | 0.00219 | 0.00137 | 0.00398 |
| 115.19086 | 0.00366 | 0.00366 | 0.00119 | 0.00358 | 0.00225 | 0.00258 | 0.00353 |
| 120.272 | 0.002 | 9.93078E-4 | 9.54859E-4 | 0.00149 | 6.77899E-4 | 0.00275 | 0.00291 |
| 125.08571 | 0.00168 | 0.00219 | 0.00234 | 0.00218 | 7.41813E-4 | 0.0039 | 0.00106 |
| 130.07771 | 0.00211 | 0.00325 | 0.00309 | 0.003 | 0.00127 | 0.00346 | 0.00355 |
| 135.248 | 0.00566 | 9.46008E-4 | 8.48336E-4 | 0.00352 | 0.00142 | 0.00249 | 0.00272 |
| 140.07657 | 0.00211 | 0.00426 | 0.00428 | 0.00105 | 0.00306 | 0.00138 | 0.00198 |
| 145.24686 | 0.00298 | 0.00229 | 0.00127 | 0.00188 | 0.00185 | 0.00341 | 0.00597 |
| 150.14971 | 0.00475 | 0.00197 | 0.00185 | 0.0026 | 0.00136 | 0.00237 | 0.00283 |
| 155.14171 | 8.90945E-4 | 0.00187 | 0.00181 | 0.00192 | 0.00153 | 0.00231 | 0.0049 |

**Fig.11 Temperature variation of flame axis**

**(a)**

|  | ΔT/℃ | | | | | | |
| --- | --- | --- | --- | --- | --- | --- | --- |
| H/mm | 1m/s | 1.5m/s | 2m/s | 2.5m/s | 3m/s | 3.5m/s | 4m/s |
| 800 | 5.74 | 11.89 | 15.48 | 15.73 | 16.12 | 16.21 | 16.24 |
| 600 | 7.8 | 15.31 | 19.39 | 22.21 | 24.35 | 24.39 | 25.19 |
| 400 | 0.01 | 5.42 | 7.78 | 12.15 | 15.4 | 15.12 | 14.93 |
| 200 | 0.67 | 1.35 | 0.45 | 2.78 | 4.09 | 3.31 | 4.01 |

**(b)**

|  | ΔT/℃ | | | | | | |
| --- | --- | --- | --- | --- | --- | --- | --- |
| H/mm | 1m/s | 1.5m/s | 2m/s | 2.5m/s | 3m/s | 3.5m/s | 4m/s |
| 800 | 4.61 | 5.13 | 5.52 | 5.84 | 6.01 | 5.97 | 6.01 |
| 600 | 4.75 | 4.73 | 4.64 | 4.82 | 4.77 | 5.01 | 5.25 |
| 400 | 1.45 | 1.78 | 1.17 | 1.62 | 2.72 | 2.33 | 1.61 |
| 200 | 0.46 | 0.51 | 0.47 | 0.46 | 0.47 | 0.73 | 0.97 |

**(c)**

|  | ΔT/℃ | | | | | | |
| --- | --- | --- | --- | --- | --- | --- | --- |
| H/mm | 1m/s | 1.5m/s | 2m/s | 2.5m/s | 3m/s | 3.5m/s | 4m/s |
| 800 | 13.14 | 14.77 | 14.94 | 15.23 | 16.54 | 16.27 | 16.3 |
| 600 | 10.34 | 13.86 | 14.99 | 15.09 | 15.13 | 16.01 | 16.71 |
| 400 | 1.3 | 1.53 | 1.74 | 2.97 | 4.04 | 5.02 | 5.97 |
| 200 | 0.04 | 0.09 | 0.15 | 1.08 | 1.65 | 1.57 | 1.51 |

**Fig.12 Fuel quality change rule**

| t | 1.0m/s | 1.5m/s | 2.0m/s | 2.5m/s | 3.0m/s | 3.5m/s | 4.0m/s |
| --- | --- | --- | --- | --- | --- | --- | --- |
| 5 | 98.5 | 98.3 | 98 | 98.1 | 98 | 98.05 | 98 |
| 10 | 96.9 | 96.6 | 96 | 96.07 | 96.35 | 96.56 | 96.85 |
| 15 | 95.16 | 94.56 | 93.8 | 93.9 | 93.97 | 94.27 | 95 |
| 20 | 92.65 | 92.65 | 91.75 | 92.65 | 92.35 | 93.55 | 94 |
| 25 | 90 | 90 | 90 | 90.1 | 90 | 91.1 | 92 |
| 30 | 88.12 | 88.12 | 88 | 89.2 | 88 | 89.3 | 91 |
| 35 | 86.1 | 86.51 | 86 | 86.7 | 86 | 87.4 | 90 |
| 40 | 84.25 | 83.85 | 84.5 | 84.7 | 83.75 | 86.2 | 88 |
| 45 | 83 | 82.77 | 83.25 | 83.25 | 82.1 | 84.7 | 86.25 |
| 50 | 82.05 | 81.05 | 81.35 | 81.12 | 81 | 82.9 | 84.3 |
| 55 | 81.1 | 80.2 | 80 | 80.1 | 80 | 81.5 | 82 |
| 60 | 80 | 79.6 | 78 | 77.99 | 77.68 | 78.7 | 80 |
| 65 | 77.34 | 76.34 | 76.25 | 76.15 | 76.02 | 77.2 | 78.25 |
| 70 | 73.95 | 74.95 | 74.56 | 74.06 | 74.03 | 75.6 | 77.5 |
| 75 | 70 | 71.7 | 72.9 | 73.2 | 72.3 | 73.3 | 74.8 |
| 80 | 67 | 70.1 | 70 | 70.9 | 71.25 | 72.1 | 72.5 |
| 85 | 66 | 68.3 | 67.68 | 69.68 | 70 | 70.1 | 70 |
| 90 | 63.05 | 65.05 | 66.02 | 66.32 | 67 | 68.1 | 69 |
| 95 | 62.15 | 63.15 | 64.35 | 65.15 | 65.5 | 65.7 | 66.5 |
| 100 | 60 | 61.2 | 62.72 | 62.82 | 62 | 63.6 | 65.55 |
| 105 | 59.68 | 60.68 | 61.04 | 61.24 | 60 | 61 | 62 |
| 110 | 56.02 | 58.02 | 60 | 59.81 | 59 | 59.7 | 60 |
| 115 | 55.36 | 56.76 | 57 | 56.9 | 55 | 56.7 | 56.3 |
| 120 | 52 | 54.1 | 56.8 | 54.8 | 54.76 | 54.1 | 53.01 |
| 125 | 51.04 | 52.69 | 55 | 53.6 | 52.3 | 51.5 | 50 |
| 130 | 50 | 51.7 | 52.05 | 52.15 | 50 | 48.4 | 47 |
| 135 | 48.56 | 49.56 | 50 | 49.9 | 47 | 46.1 | 45.25 |
| 140 | 45.5 | 47.5 | 48.5 | 47.5 | 46.05 | 43.9 | 41.2 |
| 145 | 44.78 | 45.98 | 45 | 45.89 | 45.25 | 40.7 | 40 |
| 150 | 43.05 | 43.66 | 44.25 | 43.25 | 41.5 | 37.9 | 37 |
| 155 | 40 | 42.2 | 41 | 41.1 | 40 | 36.3 | 35.25 |
| 160 | 37 | 39.4 | 40 | 39.9 | 38.15 | 33.9 | 32 |
| 165 | 35.5 | 37.77 | 37 | 37.3 | 35.5 | 31.9 | 30 |
| 170 | 32 | 34.12 | 36.15 | 35.15 | 32 | 28.8 | 27.68 |
| 175 | 30 | 32.11 | 33 | 32.23 | 31.25 | 27.3 | 26.5 |
| 180 | 28.68 | 29.48 | 32.5 | 30.5 | 30 | 26.1 | 24.36 |
| 185 | 26.02 | 27.08 | 30 | 28.7 | 28.68 | 24.8 | 23.7 |
| 190 | 25.36 | 25.86 | 27 | 26.8 | 26.02 | 22.9 | 21.01 |
| 195 | 23.7 | 24.33 | 26.5 | 25.5 | 25.36 | 20.7 | 20 |
| 200 | 21.04 | 22.67 | 25.6 | 23.62 | 23.7 | 19.4 | 18.16 |
| 205 | 20 | 21.98 | 22 | 22.1 | 21.04 | 18.7 | 17.74 |
| 210 | 18.78 | 19.98 | 20 | 20.23 | 20 | 16.8 | 15.32 |
| 215 | 17.67 | 18.97 | 19.12 | 19.82 | 18.78 | 15.1 | 14.5 |
| 220 | 16.56 | 17.86 | 18 | 18.1 | 17 | 14.2 | 12.48 |
| 225 | 15.5 | 16.99 | 17.5 | 17.1 | 16.5 | 12.9 | 11.06 |
| 230 | 14.34 | 15.84 | 16 | 16.2 | 15.45 | 11.2 | 10 |
| 235 | 13.23 | 14.83 | 15.8 | 15.8 | 14.08 | 10.3 | 8.5 |
| 240 | 12.12 | 13.52 | 14.78 | 14.68 | 13.3 | 8.9 | 6.6 |
| 245 | 11.05 | 12.65 | 13.5 | 13.65 | 12.5 | 7.7 | 5.2 |
| 250 | 10 | 11.38 | 12.18 | 12.48 | 11.5 | 6.5 | 4.1 |
| 255 | 8.5 | 10.03 | 11.15 | 11.35 | 10 | 4.9 | 3 |
| 260 | 7.5 | 9.01 | 10 | 10.32 | 9.3 | 3.4 | 2.2 |
| 265 | 5.85 | 7.88 | 8.8 | 8.78 | 8.6 | 2.7 | 1 |
| 270 | 4.25 | 6.25 | 7.5 | 7.75 | 7.57 | 1.8 |  |
| 275 | 2.5 | 5.05 | 5.65 | 6.65 | 6.95 | 1 |  |
| 280 | 1.5 | 3.25 | 3.5 | 5.15 | 5.6 |  |  |
| 285 |  | 1.54 | 1.6 | 4.06 | 4.25 |  |  |
| 290 |  |  |  | 3.01 | 3.2 |  |  |
| 295 |  |  |  | 1.48 | 2 |  |  |
| 300 |  |  |  |  | 1 |  |  |

**Fig.14 Grid independence verification of v=0m/s**

**(a)**

| t/s | 0.1m | 0.5m | 1.0m | 1.5m | 2.0m |
| --- | --- | --- | --- | --- | --- |
| 10 | 32 | 33 | 33 | 37 | 43 |
| 20 | 79 | 81 | 80 | 86 | 100 |
| 30 | 110 | 111 | 113 | 116 | 132 |
| 40 | 157 | 157 | 156 | 159 | 147 |
| 50 | 196 | 193 | 195 | 177 | 178 |
| 60 | 290 | 287 | 288 | 281 | 279 |
| 70 | 412 | 400 | 402 | 389 | 376 |
| 80 | 503 | 504 | 501 | 501 | 465 |
| 90 | 617 | 603 | 601 | 590 | 567 |
| 100 | 702 | 700 | 700 | 675 | 677 |
| 110 | 707 | 695 | 694 | 678 | 681 |
| 120 | 719 | 706 | 717 | 700 | 702 |
| 130 | 737 | 728 | 725 | 717 | 705 |
| 140 | 727 | 727 | 729 | 703 | 712 |
| 150 | 719 | 716 | 711 | 701 | 711 |
| 160 | 732 | 730 | 721 | 706 | 717 |
| 170 | 724 | 710 | 710 | 712 | 709 |
| 180 | 735 | 723 | 720 | 732 | 722 |
| 190 | 717 | 706 | 705 | 701 | 699 |
| 200 | 706 | 704 | 704 | 689 | 700 |
| 210 | 706 | 701 | 699 | 689 | 678 |
| 220 | 717 | 715 | 719 | 700 | 666 |
| 230 | 701 | 687 | 687 | 667 | 677 |
| 240 | 689 | 690 | 688 | 677 | 659 |
| 250 | 692 | 690 | 701 | 679 | 665 |
| 260 | 676 | 680 | 670 | 670 | 676 |
| 270 | 671 | 673 | 666 | 656 | 645 |
| 280 | 647 | 646 | 641 | 642 | 613 |
| 290 | 645 | 640 | 634 | 634 | 612 |
| 300 | 631 | 632 | 627 | 611 | 602 |

**(b)**

| t/s | 0.1m | 0.5m | 1.0m | 1.5m | 2.0m |
| --- | --- | --- | --- | --- | --- |
| 10 | 17 | 18 | 18 | 22 | 28 |
| 20 | 64 | 61 | 65 | 69 | 90 |
| 30 | 95 | 91 | 89 | 90 | 103 |
| 40 | 142 | 142 | 141 | 144 | 132 |
| 50 | 181 | 170 | 173 | 199 | 189 |
| 60 | 275 | 270 | 265 | 286 | 264 |
| 70 | 397 | 385 | 387 | 374 | 361 |
| 80 | 490 | 497 | 496 | 472 | 450 |
| 90 | 602 | 590 | 590 | 575 | 552 |
| 100 | 687 | 685 | 685 | 660 | 662 |
| 110 | 692 | 680 | 679 | 663 | 666 |
| 120 | 704 | 691 | 689 | 685 | 680 |
| 130 | 722 | 713 | 710 | 698 | 690 |
| 140 | 712 | 712 | 714 | 688 | 697 |
| 150 | 704 | 691 | 689 | 688 | 690 |
| 160 | 717 | 701 | 706 | 691 | 702 |
| 170 | 709 | 695 | 695 | 690 | 689 |
| 180 | 720 | 708 | 705 | 698 | 699 |
| 190 | 702 | 691 | 690 | 676 | 666 |
| 200 | 691 | 674 | 667 | 645 | 653 |
| 210 | 691 | 686 | 684 | 674 | 663 |
| 220 | 702 | 700 | 699 | 685 | 651 |
| 230 | 686 | 672 | 672 | 652 | 662 |
| 240 | 674 | 675 | 673 | 656 | 644 |
| 250 | 677 | 675 | 686 | 664 | 650 |
| 260 | 661 | 665 | 655 | 655 | 661 |
| 270 | 656 | 658 | 651 | 641 | 630 |
| 280 | 632 | 624 | 626 | 627 | 598 |
| 290 | 630 | 618 | 619 | 601 | 597 |
| 300 | 616 | 617 | 612 | 596 | 587 |

**Fig.15 Result verification**

| v | Tn | error | Qn | error |
| --- | --- | --- | --- | --- |
| 1 | 709.6 | 0.7 | 748000 | 8200 |
| 1.5 | 737.2 | 4.7 | 747700 | 1200 |
| 2 | 730.2 | 4.6 | 719000 | 2800 |
| 2.5 | 717.8 | 5 | 729800 | 190 |
| 3 | 741.1 | 9.5 | 694000 | 1900 |
| 3.5 | 707.9 | 2.4 | 758900 | 1600 |
| 4 | 727.8 | 4 | 793000 | 1900 |
